# Distinct Cyclosporin A Doses Are Required to Enhance Bone Formation Induced by Cyclic and Rest-Inserted Loading in the Senescent Skeleton

**DOI:** 10.1371/journal.pone.0084868

**Published:** 2014-01-03

**Authors:** Sundar Srinivasan, Dewayne Threet, Leah E. Worton, Brandon J. Ausk, Steven D. Bain, Edith M. Gardiner, Ronald Y. Kwon, Ted S. Gross

**Affiliations:** Department of Orthopaedics and Sports Medicine, University of Washington, Seattle, Washington, United States of America; Faculdade de Medicina Dentária, Universidade do Porto, Portugal

## Abstract

Age-related decline in periosteal adaptation negatively impacts the ability to utilize exercise to enhance bone mass and strength in the elderly. We recently observed that in senescent animals subject to cyclically applied loading, supplementation with Cyclosporin A (CsA) substantially enhanced the periosteal bone formation rates to levels observed in young animals. We therefore speculated that if the CsA supplement could enhance bone response to a variety of types of mechanical stimuli, this approach could readily provide the means to expand the range of mild stimuli that are robustly osteogenic at senescence. Here, we specifically hypothesized that a given CsA supplement would enhance bone formation induced in the senescent skeleton by both cyclic (1-Hz) and rest-inserted loading (wherein a 10-s unloaded rest interval is inserted between each load cycle). To examine this hypothesis, the right tibiae of senescent female C57BL/6 mice (22 Mo) were subjected to cyclic or rest-inserted loading supplemented with CsA at 3.0 mg/kg. As previously, we initially found that while the periosteal bone formation rate (p.BFR) induced by cyclic loading was enhanced when supplemented with 3.0 mg/kg CsA (by 140%), the response to rest-inserted loading was not augmented at this CsA dosage. In follow-up experiments, we observed that while a 30-fold lower CsA dosage (0.1 mg/kg) significantly enhanced p.BFR induced by rest-inserted loading (by 102%), it was ineffective as a supplement with cyclic loading. Additional experiments and statistical analysis confirmed that the dose-response relations were significantly different for cyclic versus rest-inserted loading, only because the two stimuli required distinct CsA dosages for efficacy. While not anticipated *a priori*, clarifying the complexity underlying the observed interaction between CsA dosage and loading type holds potential for insight into how bone response to a broad range of mechanical stimuli may be substantially enhanced in the senescent skeleton.

## Introduction

Age-related bone loss markedly compromises skeletal integrity and increases fracture risk [1], [2]. While interventions against osteoclastic bone resorption have proved successful [3], anabolic therapies that can initiate *de novo* bone formation and thereby compensate for accumulated loss of bone mass over age are currently under consideration (e.g., PTH, sclerostin antibodies) [4]. Physical exercise and mechanical loading are also anabolic for bone and hold therapeutic potential to focally buttress weakened skeletal structures [5], particularly given the ability of mechanical loading to substantially influence bone size via periosteal expansion [6]. However, when exercise based trials have been applied to the elderly, they have proven ineffective in influencing bone mass and strength [7]. While many differences exist between the aged and young skeleton, two factors clearly hold potential to limit the effectiveness of exercise in the elderly: 1) age-related alterations in cell function [8], [9], [10], [11], [12], and/or 2) poor compliance with the high-intensity types of exercise most capable of periosteal expansion [13], [14].

Pre-clinical animal models have unique potential to explore age-related mechanotransduction deficits. However, there is controversy in the literature regarding the degree to which aging alters the responsiveness of the periosteal surface to mechanical stimuli [15], [16], [17], [18]. We have previously reviewed this issue extensively [19], and under controlled conditions, aged animal models can be used to explore the muted periosteal adaptation to physical exercise typically encountered in the elderly [15], [16]. For example, our studies in senescent C57BL/6 mice (22 Mo) suggest that bone adaptation to mechanical stimuli is substantially decreased versus that in young [20], and attempts to enhance the low-level bone formation by doubling strain magnitude, increasing cycle numbers and rest-insertion were all ineffective in the senescent skeleton [21]. In contrast, bone formation is differentially enhanced by each of these [22] and other types of perturbations of a ‘control’ mechanical stimulus in young animals [23], [24], [25], [26]. The consequence of this loss of differential tissue responses in the context of low-level periosteal adaptation is a marked reduction in the range of mild, readily complied with mechanical stimuli options capable of robustly influencing periosteal adaptation in the senescent skeleton.

Recently, in a computational model of acute signaling pathway activation during mechanical loading, we observed that specific deficits in activation and nuclear binding of transcription factors were sufficient to account for the muted response of the senescent periosteum to a variety of mechanical stimuli [20]. Based upon model derived insights, we performed a follow-up experiment in which senescent animals were subjected to cyclically applied mechanical stimuli with or without low dose Cyclosporin A (CsA; either 0.3 or 3.0 mg/kg). We found that when cyclical loading was supplemented with CsA, the ensuing bone formation was substantially increased and equivalent to that induced in young animals by loading alone [20].

Given this outcome, we speculated that supplementation with CsA could substantially enhance bone formation induced in senescent animals by different types of mechanical stimuli. As a starting point, we specifically hypothesized that a given CsA supplement would enhance bone formation induced in the senescent skeleton by both cyclic (1-Hz) and rest-inserted loading (wherein a 10-s unloaded rest is inserted between each load cycle). We examined our hypothesis by subjecting the right tibiae of senescent female C57BL/6 mice (22 Mo) to cyclic or rest-inserted loading supplemented with CsA at a dosage (3.0 mg/kg) previously found to be maximally efficacious [20]. However, based upon findings contrary to expectations, we were then compelled to explore the possibility that the two different mechanical loading stimuli (i.e., cyclic, rest-inserted) required distinct CsA dosages in order to significantly enhance bone formation response in senescent animals.

## Methods

### Ethics Statement

All experiments and protocols were approved by the University of Washington, Institutional Animal Care and Use Committee (IACUC 3306-02).

### Methods Overview

All together, we performed a series of three experiments in this study. In each experiment, senescent female C57BL/6 mice (22 Mo; obtained from NIH aged rodent colonies) were subject to mechanical loading with or without CsA supplementation. Mechanical loading was exogenously applied to the right tibiae of anesthetized mice (2% isoflurane) using the noninvasive murine tibia-loading device [27]. Animals were subject to 50 cycles/d of mechanical loading on M, W, F for 3 weeks (9 total bouts), with protocols calibrated to induce peak longitudinal normal strain of 1700 µε at the tibia mid-shaft. The loading was applied as either a cyclic (1-Hz) or a rest-inserted waveform (10-s unloaded rest between each load cycle). Prior to the experiments, the right tibia mid-shaft was scanned via *in vivo* microCT (SCANCO vivaCT-40), and animal specific longitudinal normal strains induced by the loading protocols were determined for individual animal mid-shaft cross-sections via beam theory [22]. In addition, animals received CsA supplements s.c. 30 mins prior to each loading bout (Sandimmune, Novartis Pharma), at concentrations specified within each experimental design. Stock solutions were prepared at different dilutions (in physiologic saline), such that experimentally specified concentrations (e.g., 3.0 mg/kg) were re-constituted within each 200 µl s.c. injection (dilutions were prepared by assuming an average mouse weight of 28 gm).

Animal weights were measured prior to loading/CsA administration on each of 9 loading days. Animals were permitted food and water ad libitum. All animals received Calcein labeling i.p. (10 mg/kg) on day 10, 19, and were sacrificed on day 22. Upon sacrifice, the right loaded and left contralateral tibia were dissected of soft tissue and 300 µm thick sections were obtained about the tibia mid-shaft. The sections were hand ground to 90 µm, cover slipped and imaged via an epifluorescent microscope (Nikon, 20x). As previously [22], composite images were assembled, anatomically oriented and standard dynamic histomorphometry measures of mineral apposition rate (MAR, µm/d), surface referent mineralizing surface (MS/BS%) and bone formation rate (BFR, µm^3^/µm^2^/d) were determined at the periosteal surface using custom software within Image J.

#### Experiment 1

We first performed an experiment in order to examine our hypothesis that a given CsA supplement would enhance bone formation induced in the senescent skeleton by both cyclic (1-Hz) and rest-inserted loading. For this, the right tibiae of senescent female mice were exposed to cyclic or rest-inserted mechanical loading with/without CsA supplementation. Separate groups of senescent animals received cyclically applied loading with CsA supplementation at 0.0 mg/kg (n = 9), or 3.0 mg/kg (n = 10), or rest-inserted loading supplemented with CsA at 0.0 mg/kg (n = 8) or 3.0 mg/kg (n = 7). One (out of 10) animal in the cyclic loading group supplemented with 3.0 mg/kg died during the experiment and was not included in the analysis.

#### Experiment 2

Based upon the outcomes of Experiment 1, and a synthesis of the literature on the mechanisms of action of CsA and putative interactions with mechanotransduction pathways, we performed a second experiment. We explored the possibility that rest-inserted loading required a lower dose CsA supplement in order to enhance loading induced bone formation. For this, senescent C57BL/6 mice were exposed to rest-inserted loading supplemented with CsA at 0.1 mg/kg (n = 9) or 0.3 mg/kg (n = 7). As comparative controls, animals were also exposed to cyclic loading supplemented with CsA at 0.1 mg/kg (n = 10), or 0.3 mg/kg (n = 8). Lastly, to examine whether CsA supplementation enhanced bone formation versus that induced by loading alone, and to minimize use of experimental animals, the data from animals receiving loading but not CsA from Experiment 1 was also included in the analysis. One animal each that was exposed to cyclic loading supplemented with CsA at 0.1 mg/kg and 0.3 mg/kg died during the experiment and were therefore not included in the analysis.

#### Experiment 3

The results of Experiments 1 and 2 suggested the possibility of a different dose response relation for rest-inserted vs cyclic stimuli when supplemented with CsA. Specifically, it appeared that response to cyclic loading supplemented with CsA could be linear, while response to rest-inserted loading supplemented with CsA could be bi-phasic. To more rigorously confirm this possibility while minimizing use of senescent experimental animals, we included two additional experimental groups where CsA dosages were biased based upon the loading waveform implemented (i.e., higher dose for cyclic and lower dose for rest-inserted loading than those previously implemented in Experiments 1, 2). Specifically, a group of animals were exposed to cyclic loading supplemented with CsA at 6.0 mg/kg (n = 8). A separate group of animals (n = 7) were subject to rest-inserted loading and supplemented with 0.03 mg/kg CsA. The data from Experiments 1 and 2 were included along with the data generated in Experiment 3 for analysis of dose-response relations. One animal exposed to cyclic loading supplemented with CsA at 6.0 mg/kg died prematurely and was therefore not included in the analysis.

To analyze whether the dose-response relation was significantly different for cyclic vs rest-inserted loading, we determined relative measures of periosteal bone response. Specifically, we determined relative periosteal MS/BS (rp.MS/BS), MAR (rp.MAR), and BFR (rp.BFR) as responses measured in the experimentally loaded – contralateral limbs. Given that the consolidated data appeared to follow a biphasic pattern, we developed a non-linear model, similar in functionality to hormesis models [28], [29], [30], to explore and analyze the dose-response relation. Specifically, we implemented a model of the form:
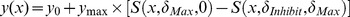
(1a)where,



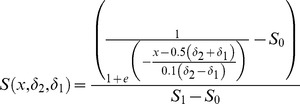
(1b)

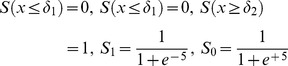
(1c)and, ‘*y*’ is the bone response measure (i.e., rp.MS, rp.MAR, or rp.BFR), and ‘*x*’ is the CsA dosage in mg/kg. Each of the implemented parameters of the current model (n = 4) had a biological relevance. The four parameters modeled the baseline response induced by loading in the absence of CsA (*y_0_*), the maximal quantity by which the response is increased beyond baseline (*y_Max_*) when loading is supplemented with the optimal dosage of CsA *(δ_Max_*), and the dosage at which the response ‘y’ returns to baseline *(δ_Inhibit_*). This model then permitted us to examine whether the dose-response relations (and aspects within) were equivalent or not between cyclic and rest-inserted loading supplemented with CsA.

### Statistical Analysis

Data were primarily analyzed via factorial MANOVA with cyclic vs rest-inserted loading, experimentally loaded vs contralateral tibiae, and CsA dosage as independent factors and with MS/BS, MAR and BFR at the periosteal surface as the dependent variates. Where main effects were significant (and post-hoc analysis required), Dunnett’s control test were performed to examine whether CsA supplementation significantly enhanced bone formation compared to that induced by loading alone. All test were performed using SPSS statistical software, and p≤0.05 was considered to be statistically significant.

To explore dose-response relations, we calibrated the parameters of the non-linear model (eq 1) against the *in vivo* relative bone response measures (rp.MS, rp.MAR, rp.BFR). For this, we used a previously implemented method of maximum likelihood estimation (MLE) and likelihood ratio testing (LRT) [20], [31]. Briefly, the method of MLE attempts to determine the model parameters that best simulate experimental data means given experimental variance. Upon parameter estimation, the approach then permits a determination of whether the model (and hence, the dose-response relations) are significantly different between cyclic and rest-inserted loading, as well as a determination of which aspects of the model contribute to the differences in dose-response relations. To implement this approach, we assumed that the data were normally distributed and maximized the likelihood estimate for the non-linear model (eq 1) using the simulated annealing algorithm (SA; 100,000 iterations with 10 random restarts). The MLE and LRT analyses were performed within MATLAB software.

Using this statistical approach, we first explored the null hypothesis that a single model (n = 4 parameters) could simulate the dose-response relation for both cyclic and rest-inserted loading supplemented with CsA. If so, we additionally examined whether constraining parameter *y_Max_ = *0 (i.e., is there a benefit with CsA administration?) permitted simulation of the experimental data. Second, for response measures where the first analysis failed, we explored the null hypothesis that 2 separate models (n = 8 parameters) could simulate the dose-response relation for cyclic and rest-inserted loading with CsA. Subsequent analyses were then performed for response measures where the second analysis was successful (i.e., the null hypothesis was accepted). Third, we explored whether null hypotheses constraining each of the 4 modeled parameters to be the same between the cyclic and rest-inserted model, when considered individually and in combinations, could be accepted or rejected. Lastly, we examined whether null hypotheses constraining parameter *y_Max_ = *0 was within the 95% confidence interval of the MLE for the cyclic and rest-inserted models. We used LRTs to either accept (p>0.05) or reject (p≤0.05) each of the null hypotheses posed above against alternate models. For the first two analyses above, the alternate model comprised a fully saturated model who’s ‘global’ maximum likelihood estimate (GMLE) was determined by assigning the model means to be equal to the experimental means for each of the experimental groups [20]. For subsequent analyses, the alternate models for null hypotheses contrasts were the MLE’s for either the 4 parameter or 8 parameter models described above [20].

## Results

Loading induced animal specific strains of groups included in Experiment 1, 2 and 3 were not significantly different across comparisons (Table 1; p≥0.2).

**Table 1:** Estimated strains (µε; mean ± s.e.) induced in animals across the CsA dosages by cyclic and rest-inserted loading.

	CsA dosage (mg/kg)					
	0.0	0.03	0.1	0.3	3.0	6.0
Cyclic	1745±47	–	1670±70	1638±94	1760±47	1848±86
Rest	1736±53	1658±65	1696±55	1813±44	1926±127	–

### Experiment 1

**Figure 1:**
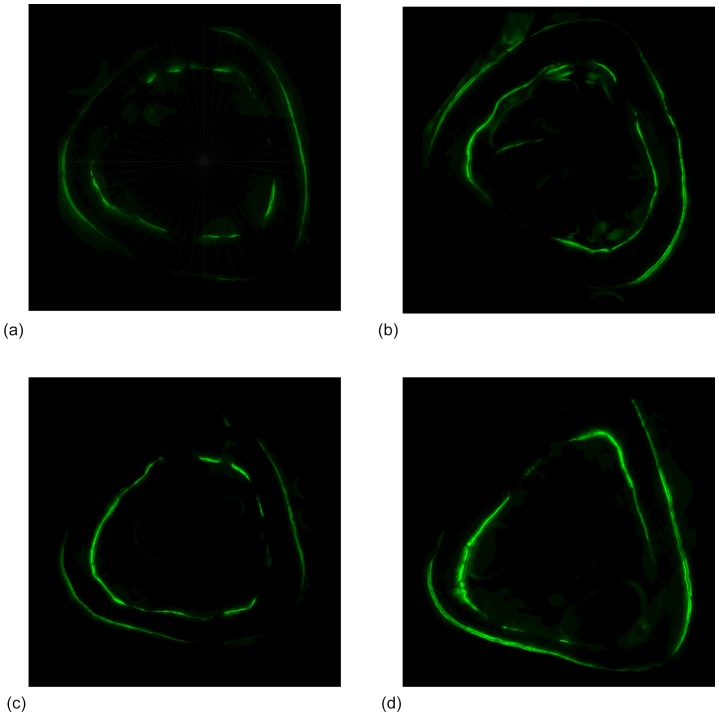
Bone response at the mid-shaft tibiae from animals subject to loading with or without CsA supplementation. Illustrative fluorescent images from animals subject to cyclic loading (a) and supplemented with 3.0 mg/kg CsA (b), or subject to rest-inserted loading (c) and supplemented with 0.1 mg/kg CsA (d).

**Figure 2:**
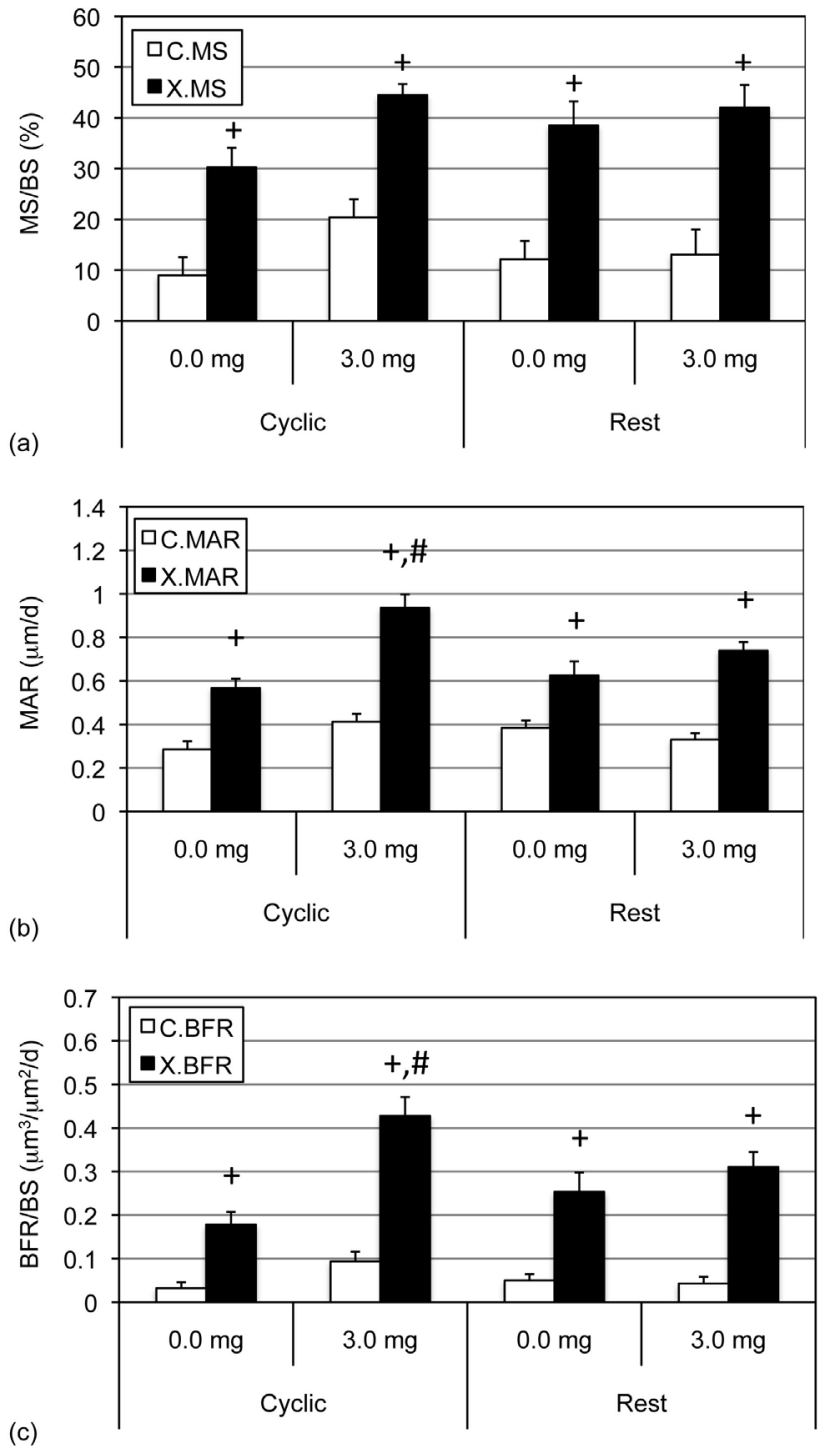
CsA supplementation significantly amplified bone response to cyclic loading. Periosteal MS/BS (a), MAR (b), and BFR/BS (c; mean+s.e.) were significantly increased in experimentally loaded (X) versus contralateral controls (C; ‘+’). Supplementing cyclic loading with CsA (at 3.0 mg/kg) significantly enhanced MAR and BFR/BS compared with that induced by loading alone (‘#’).

In our initial experiment, we sought to determine whether CsA supplementation at a previously efficacious dosage for cyclic loading (3.0 mg/kg), could also enhance bone formation induced by rest-inserted loading. We found that in the contralateral bones, the main effect of loading (cyclic, rest) or CsA did not attain significance for MS (p = 0.27), MAR (p = 0.13), and BFR (p = 0.17). In animals subject to cyclic loading MS, MAR and BFR were significantly increased in the experimentally loaded vs contralateral bones when supplemented by 0.0 mg/kg CsA (p≤0.001), and by 3.0 mg/kg CsA (p≤0.001; Fig. 1, 2). In animals subject to rest-inserted loading MS, MAR and BFR were significantly increased in the experimentally loaded vs contralateral bones when supplemented by 0.0 mg/kg CsA (p≤0.01), and by 3.0 mg/kg CsA (p≤0.001; Fig. 2).

In the experimentally loaded bones, the main effect of cyclic versus rest-inserted loading supplemented with CsA (0.0, 3.0 mg/kg) attained significance for MAR (p = 0.001) and BFR (p<0.01), but not for MS (p = 0.07; therefore, influence upon MS was not further analyzed). In the experimental limbs of cyclically loaded animals, CsA supplementation at 3.0 mg/kg significantly enhanced MAR (p = 0.001) and BFR (p<0.01) vs that induced by cyclic loading alone (Fig. 1, 2). In the experimental limbs of rest-inserted loaded animals, neither MAR nor BFR were significantly influenced by CsA supplementation (p ≥ 0.17). Lastly, the bone response parameters were not significantly different in the experimentally loaded bones of animals exposed to cyclic vs rest-inserted loading without or with CsA supplementation (p = 0.29).

### Experiment 2

**Figure 3:**
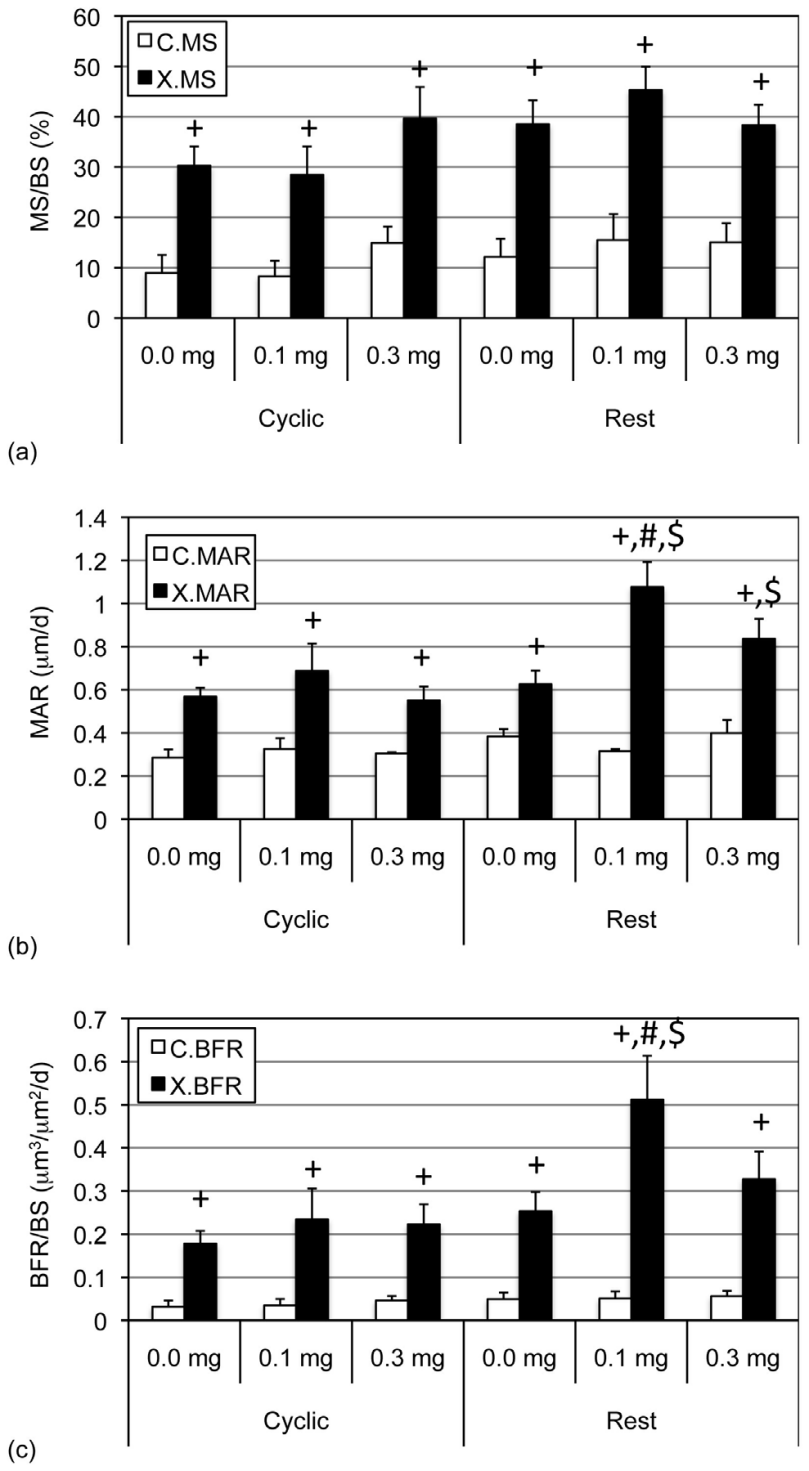
Lower-dose CsA supplementation was required to amplify bone response to rest-inserted loading. Periosteal MS/BS (a), MAR (b), and BFR/BS (c; mean+s.e.) were significantly increased in experimentally loaded (X) versus contralateral controls (C; ‘+’). Supplementing rest-inserted loading, but not cyclic loading, with low-dose CsA (at 0.1 mg/kg) significantly enhanced MAR and BFR/BS compared with that induced by rest-inserted loading alone (‘#’). Additionally, CsA supplementation enhanced MAR (at 0.1, 0.3 mg/kg) and BFR/BS (at 0.1 mg/kg) in animals subject to rest-inserted versus cyclic loading (‘$’).

We next examined whether lower dosage CsA supplements (0.1 or 0.3 mg/kg) could enhance bone formation induced by rest-inserted loading (and cyclic loading as comparative controls). We found that in the contralateral bones, the main effects of dosage (0.0 vs 0.1 vs 0.3 mg/kg CsA) or protocol type (cyclic, rest-inserted) did not alter outcome measures (p = 0.29; Fig. 3). In animals subject to cyclic loading, MS, MAR and BFR were significantly increased in the experimentally loaded vs contralateral bones when supplemented with 0.1 mg/kg CsA (p≤0.02) and by 0.3 mg/kg CsA (p≤0.01; Fig. 3). In animals subject to rest-inserted loading MS, MAR and BFR were significantly increased in the experimentally loaded vs contralateral bones when supplemented by 0.1 mg/kg CsA (p≤0.001) and by 0.3 mg/kg CsA (p≤0.01; Fig. 3).

In the experimentally loaded bones, the main effect of cyclic versus rest-inserted loading supplemented with CsA (0, 0.1, 0.3 mg/kg) attained significance for MAR (p<0.01) and BFR (p<0.01), but not for MS (p = 0.14). In bone exposed to cyclical loading, neither MAR nor BFR were significantly influenced across the CsA dosages (p ≥ 0.32; Fig. 3). In the experimental limbs of rest-inserted loaded animals, CsA supplementation at 0.1 mg/kg, but not at 0.3 mg/kg significantly enhanced MAR (p<0.01) and BFR (p = 0.02) vs that induced in animals subject to rest-inserted loading without CsA (0.0 mg/kg; Fig. 1, 3). Lastly, supplementation with CsA at 0.1 and 0.3 mg/kg CsA significantly enhanced MAR (p≤0.04) and supplementation with 0.1 mg/kg of CsA significantly enhanced BFR (p = 0.04) in animals exposed to rest-inserted vs cyclical loading (Fig. 3).

### Experiment 3

In this experiment, we included two additional groups (cyclical loading with 6.0 mg/kg and rest-inserted loading with 0.03 mg/kg CsA) alongside previous data to determine dose-response relations. In the contralateral bones, we found that the main effect of CsA supplementation and loading (cyclic, rest-inserted) upon the response measures were not significant (p = 0.33). In animals subject to cyclic loading supplemented with CsA at 6.0 mg/kg, only MAR was significantly increased in experimentally loaded vs contralateral bones (0.59±0.05 vs 0.39±0.04 µm/d, respectively; p = 0.01). In animals subject to rest-inserted loading supplemented with CsA at 0.03 mg/kg, MS (38.3±4.1 vs 15.0±3.8%; p<0.001), MAR (0.84±0.09 vs 0.40±0.06 µm/d; p<0.01) and BFR (0.33±0.06 vs 0.06±0.01 µm^3^/µm^2^/d; p = 0.001) were significantly increased in experimentally loaded vs contralateral bones, respectively.

**Figure 4:**
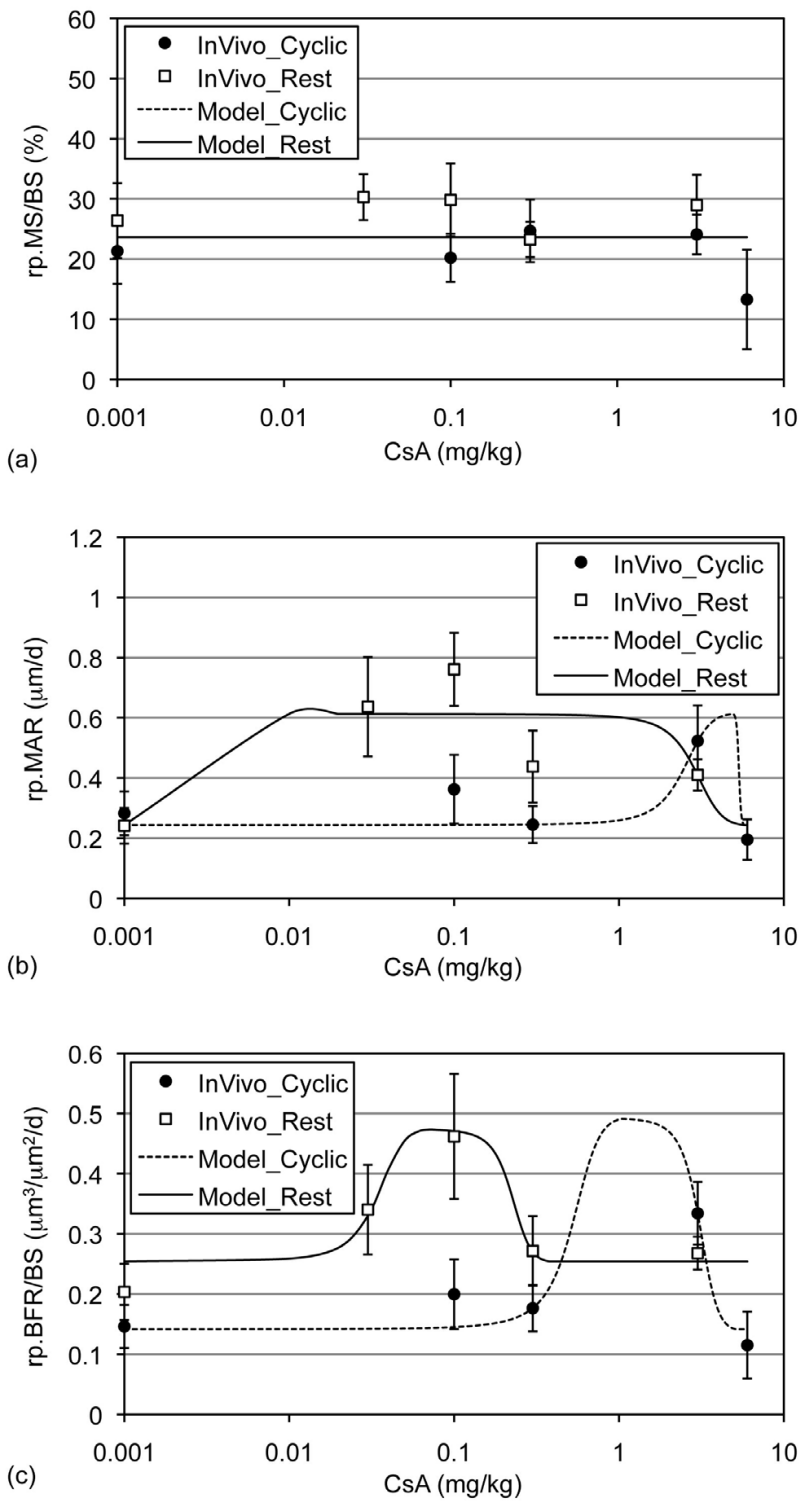
Dose-response relations simulated by the implemented non-linear models. A single model, which assumed that there is no adaptive benefit of loading supplemented with CsA, could simulate the rp.MS/BS data (a; mean ± s.e.). In contrast, two separate models were required to simulate rp.MAR (b) and rp.BFR/BS (c) induced by cyclic and rest-inserted loading supplemented with CsA. Please note that CsA dosage is plotted on a log-scale.

To explore dose-response relations, we included these data along side the data from Experiments 1 and 2. We implemented our non-linear model (eq 1) and used relative bone histomorphometry parameters as outcomes (i.e., rp.MAR, rp.MS, rp.BFR). Acceptance (or rejection) of null hypotheses describing the dose-response relations was dependent upon the assayed response. In our analysis of rp.MS, we found that a single model (i.e., parameters constrained to be the same for cyclic and rest-inserted models) including the null hypothesis that CsA supplementation has no benefit (i.e., *y_Max_ = *0) could describe the response induced by both cyclic and rest-inserted loading across the CsA dosages implemented experimentally (p = 0.32; Fig. 4; Table 2). In contrast, the single model null hypothesis was rejected for both rp.MAR (p = 0.03) and rp.BFR (p = 0.01).

**Table 2:** MLEs for model parameters for p.MS/BS, p.MAR, and p.BFR/BS (+ p<0.05 vs cyclic).

		*y_0_*	*y_Max_*	*δ_Max_*	*δ_Inhibit_*
p.MS/BS	Cyclic	23.625	0.0	5.9047	12.649
	Rest	23.625	0.0	5.9047	12.649
p.MAR	Cyclic	0.2437	0.3694	4.9215	5.7544
	Rest	0.2437	0.3694	0.0029^+^	5.7544
p.BFR	Cyclic	0.1415	0.3498	1.0444	5.1182
	Rest	0.2543	0.219	0.0686^+^	0.3789

As a result, we explored the null hypothesis that two separate models were required to simulate the rp.MAR and rp.BFR data. The null hypotheses that two separate models for cyclic and rest-inserted loading were required to describe the rp.MAR (p = 0.09) and rp.BFR response were both accepted (p = 0.25; Fig. 4; Table 2). However, the null hypothesis that CsA supplementation has no benefit (i.e., *y_Max_ = *0) as a supplement for cyclic and rest-inserted loading was rejected for both rp.MAR (p≤0.001) and rp.BFR (p≤0.03). Given these findings, further analysis of the modeled parameters suggested that the optimal dosage at which CsA maximally enhances loading induced bone formation (i.e., *δ_Max_*) was significantly different between cyclic and rest-inserted loading models (p = 0.01 for rp.MAR and p = 0.03 for rp.BFR). Null hypotheses constraining the remaining parameters to be the same between the cyclic and rest-inserted models, when considered separately and in combinations, were all accepted for both rp.MAR (p≥0.1) and rp.BFR response measures (p≥0.06).

## Discussion

The loss of differential responses and the low-level bone response to mechanical signals markedly reduces the types of mild loading stimuli that can be used to robustly influence bone adaptation at senescence. Based upon the previously observed efficacy of cyclical loading supplemented with CsA [20], we began to explore whether CsA supplementation could enhance bone formation induced by different types of mechanical stimuli in the senescent skeleton. We found that CsA supplementation substantially enhanced bone formation induced by both cyclic and rest-inserted mechanical loading, but unexpectedly required distinct dosages for each mechanical stimulus (3.0, 0.1 mg/kg, respectively). Statistical analyses confirmed that CsA supplementation of cyclic and rest-inserted loading resulted in unique dose-bone response relations only at the ‘optimal’ dosage at which enhanced adaptation was induced. Given this unexpected outcome, a mechanistic understanding of CsA – loading interactions may be required for the optimized design of protocols that enhance bone response to a broad range of mild mechanical stimuli in the senescent skeleton.

Prior to interpreting our data, we discuss some of the limitations of our study. We designed a non-linear model to explore the dose-response relation and to determine whether specific aspects of the relation were different between cyclic and rest-inserted loading supplemented with CsA. While the model was specifically designed to simulate bi-phasic data, alternate models have also been developed for this purpose [28], [29], [30]. Of note, given the resolution of the model in simulating the data, statistical analysis suggests that model refinement or alternate model implementation will not significantly improve simulation of the current data set. Secondly, we observed premature animal death (n = 4) in groups subjected to cyclic loading. We consider these adverse events to be primarily due to the advanced age of these animals, considering the absence of adverse events in animals subject to rest-inserted loading with/without CsA (a protocol that notably required a 2 fold greater duration under anesthesia), as well as a lack of significant weight change in animals across time.

Our current data were inconsistent with our previous observations in one instance [20]. Specifically, here we found that supplementing cyclical loading with CsA at 0.3 mg/kg did not significantly enhance loading induced bone formation as was previously observed (where p.BFR increased from 0.12±0.02 to 0.21±0.02 µm^3^/µm^2^/d) [20]. In the current study, while CsA supplementation at 0.3 mg/kg induced a similar p.BFR (0.22±0.05 µm^3^/µm^2^/d), the response in animals subject to cyclic loading alone was 0.18±0.03 µm^3^/µm^2^/d. Thus the difference in the studies was not in the response to the 0.3 mg/kg CsA supplement, but in the response of the aged mice to cyclically applied loading (of note, the cyclical loading protocols were implemented identically across studies and the loading induced strains in animals were also not significantly different, p = 0.56; 1745±47 vs 1703±55 µε in the previous study). While the p.BFR response to cyclic loading in the previous study was 33% lower than currently, it was within 1 S.D. of the across study means and was similar to the experimental variability observed in mice at these advanced ages [21].

In our first experiment, we observed that CsA at the dosage previously found to be maximally efficacious for cyclic loading (3.0 mg/kg) was ineffective as a supplement for rest-inserted loading. This outcome was initially disappointing; it raised the possibility that supplementing loading with CsA was efficacious only in a very limited context (of the implemented cyclic stimulus). To further explore this confounding outcome, we considered mechanisms via which CsA might interact with loading induced activation of signaling pathways. Briefly, while CsA primarily inhibits Calcineurin (Cn) activity, it has off target effects on interacting MAPKs (p38, ERK, JNK), and with GSK-3β signaling [32], [33] – competitive pathways also involved in bone mechanotransduction [34], [35], [36], [37], [38], [39]. Thus, the benefits of supplementing cyclic loading with CsA could emerge via differential suppression of Cn, JNK, p38 and GSK-3β signaling, and a resulting bi-phasic enhancement in ERK activation and AP-1 transcription [33]. However, given reports that rest-inserted stimuli enhance Ca^2+^ signaling [40], and significantly more ERK phosphorylation [41], it was likely that this stimulus would require a larger quantity of uninhibited intermediate kinases and phosphatases vs cyclic stimuli (e.g., Cn, JNK, ERK), in order for augmentation of downstream events. As such, in order for acute signaling induced by rest-inserted loading (vs cyclic loading) to be augmented by CsA, a lower quantity of ‘beneficial’ intermediates (e.g., JNK, Cn) may ‘ideally’ need to be inhibited.

This interpretation of the literature suggested the possibility that rest-inserted loading might require CsA supplementation at a lower dosage compared with cyclic stimuli. In support, our in vivo data (Fig. 1–3) and analysis (Fig. 4) confirmed that cyclic and rest-inserted loading require distinct CsA dosages. In particular, a 30-fold lower CsA dosage was required before bone formation induced by rest-inserted loading could also be substantially enhanced in senescent animals. While CsA supplementation significantly augmented responses to both cyclic and rest-inserted loading, the induced adaptation was statistically equivalent between mechanical stimuli. We believe that further protocol refinement that explicitly considers previously unanticipated interactions between CsA dosage and loading type may both augment *and* re-establish differential responses to these stimuli, as observed in young animals [25], [26], [42], [43].

However, the observed interaction between CsA dosage and mechanical stimuli substantially compounds the complexity of attempts at protocol design optimization. Theoretically, every perturbation of a ‘control’ mechanical stimulus (e.g., increased strain, load cycles, frequency, rest-insertion) may require a unique optimal drug dosage that must be experimentally confirmed. Additionally, given that different types of mechanical stimuli may differentially activate mechanotransduction signaling pathways [5], [41], other agents that interact with mechanical stimuli (e.g., PTH, IGF-1) may also exhibit this complexity [27], [44]. In this case, alternate approaches such as utilizing mechanistic computational modeling may enable exploration and optimization of such interactions *in silico*, prior to examination of a limited sub-set of protocols in animal studies [20].

In conclusion, periosteal bone response to cyclical and rest-inserted loading induced in senescent animals was significantly enhanced when supplemented with distinct dosages of Cyclosporin A. Despite development as an immunosuppressive agent, the effects of CsA are bi-phasic and at low dosages, CsA has been shown to down regulate osteoblast proliferation while increasing differentiation and mineralization [33]. Consistent with this finding, in our studies, CsA enhances bone formation primarily by increasing mineral apposition rates. Taken together, the effects of CsA in enhancing bone formation (while reducing TRAP-positive osteoclasts) in the trabecular compartment [33], and its ability to enhance loading induced bone formation in the cortical compartment of senescent animals suggest new uses for this in-expensive drug [45]. Given the potential for interaction between systemic drugs and mechanotransduction as observed here, locating optimal effective drug concentrations and supplementation regimens may require implementation of more complex experimental designs. We speculate that approaches that integrate experimental studies with computational tools may be required for the discovery of optimized protocols that enable the deployment of a broad range of mild, yet potently osteogenic regimens in the senescent skeleton.
